# Predicting Prognosis of Intracerebral Hemorrhage (ICH): Performance of ICH Score Is Not Improved by Adding Oral Anticoagulant Use

**DOI:** 10.3389/fneur.2018.00100

**Published:** 2018-02-28

**Authors:** Rik Houben, Floris H. B. M. Schreuder, Kim J. Bekelaar, Danny Claessens, Robert J. van Oostenbrugge, Julie Staals

**Affiliations:** ^1^Department of Neurology, Maastricht University Medical Centre, Maastricht, Netherlands; ^2^Department of Neurology, Radboud University Medical Centre, Nijmegen, Netherlands; ^3^Cardiovascular Research Institute Maastricht, Maastricht University Medical Centre, Maastricht, Netherlands

**Keywords:** mortality, intracerebral hemorrhage, oral anticoagulants, intracerebral hemorrhage score, prognosis

## Abstract

**Background:**

The intracerebral hemorrhage (ICH) score is a commonly used prognostic model for 30-day mortality in ICH, based on five independent predictors (ICH volume, location, Glasgow Coma Scale, age, and intraventricular extension). Use of oral anticoagulants (OAC) is also associated with mortality but was not considered in the ICH score. We investigated (a) whether the predictive performance of ICH score is similar in OAC-ICH and non-OAC-ICH and (b) whether addition of OAC use to the ICH score increases the prognostic performance of the score.

**Methods:**

We retrospectively selected all consecutive adult non-traumatic ICH cases (three hospitals, region South-Limburg, the Netherlands 2004–2009). Mortality at 30 days was recorded. Using univariable and multivariable logistic regression, association between OAC use and 30-day mortality was tested. Then (a) we computed receiver operating characteristic (ROC) curves for ICH score and determined the area under the curve (AUC) in OAC-ICH and non-OAC-ICH. Then (b) we created a New ICH score by adding OAC use to the ICH score. We calculated correlation between 30-day mortality and ICH score, respectively, New ICH score using Spearman correlation test. We computed ROC curves and calculated the AUC.

**Results:**

We analyzed 1,232 cases, 282 (22.9%) were OAC related ICH. Overall, 30-day mortality was 39.3%. OAC use was independently associated with 30-day mortality (OR 2.09, 95% CI, 1.48–2.95; *p* < 0.001), corrected for the five predictors of the ICH score. The ICH score performed slightly better in non-OAC-ICH (AUC 0.840) than in OAC-ICH (AUC 0.816), but this difference was not significant (*p* = 0.39). The ICH score and the New ICH score were both significantly correlated with 30-day mortality (rho 0.58, *p* < 0.001 and 0.59, *p* < 0.001, respectively). The AUC for the ICH score was 0.837, for New ICH score 0.840. This difference was not significant.

**Conclusion:**

The ICH score is a useful tool for predicting 30-day mortality both in patient who use and patients who do not use OAC. Although OAC use is an independent predictor of 30-day mortality, addition of OAC use to the existing ICH score does not increase the prognostic performance of this score.

## Introduction

Spontaneous non-traumatic intracerebral hemorrhage (ICH) constitutes about 10–15% of all strokes and has a high mortality of approximately 40% at 1 month (1). To make treatment decisions and being able to determine a prognosis, it is important to know which factors predict outcome. Several prediction models have been developed (2–8); the most widely used is the ICH score (3). Independent predictors for 30-day mortality in the ICH score are greater ICH volume, infratentorial location of ICH, low score on Glasgow Coma Scale (GCS), older age, and intraventricular extension of the hemorrhage.

Up to 25% of all ICH is associated with the use of oral anticoagulants (OAC), and this percentage increases with age (9). OAC use is associated with larger ICH volume, more hematoma expansion, and more intraventricular expansion (3, 10–12). Mortality in ICH associated with OAC use (OAC-ICH) is higher than in non-OAC-ICH (13, 14). However, most prognostic models, including ICH score, did not consider or include OAC use. Consequently, it is not unequivocally known whether OAC use is an additional prognostic factor, independent of the factors in the ICH score that are known to have prognostic value. Furthermore, whether the predictive performance of ICH score is similar in OAC-ICH and non-OAC-ICH has only been investigated in a recent small single-center study (15).

Therefore, we aim to determine the prognostic performance of ICH score for 30-day mortality in OAC-ICH and non-OAC-ICH in a large retrospectively collected multi-center cohort of ICH patients. We also aim to test whether OAC use is an independent predictor of outcome in ICH patients and if addition of OAC use to the ICH score increases the prognostic performance of the score.

## Materials and Methods

### Patient Selection

We retrospectively collected all consecutive adult (≥18 years) patients with an imaging-confirmed (CT- or MRI-scan) non-traumatic ICH, seen in the emergency department, inpatient or outpatient clinic, in three hospitals of South-Limburg, the Netherlands, from January 2004 to December 2009. Patients were selected using diagnosis-treatment codes retrieved from hospital Medical Registration Archives complemented with hospital stroke registries. Recurrent ICH cases were included.

Exclusion criteria were traumatic ICH or non-parenchymal hemorrhage (e.g., primary subdural, epidural, subarachnoid, or intraventricular hemorrhage), hemorrhagic transformation of ischemic stroke, brain tumor associated hemorrhage, or hemorrhage with a known vascular malformation. Patients with non-accessible charts and/or scans were also excluded.

The medical ethical committee of Maastricht University Medical Center approved the study.

### Variables and Definitions

We recorded age, sex, GCS at admission, current OAC use, and first INR at admission. OAC-ICH was defined as the occurrence of an ICH while on treatment with oral vitamin K antagonists at admission. Hemorrhage properties were measured on first CT- or MRI-scan: supra- or infratentorial, intraventricular extension, and ICH volume. ICH volume was calculated using the ABC/2 method (16, 17). Survival status and date of death was checked in 2016 in the hospital registry and the Dutch Municipal Personal Records Database. Outcome was defined as mortality at 30 days.

### ICH Score

The ICH score (0–6) was calculated as described by Hemphill et al. (3). One point was given for age >80 years, one point for infratentorial origin, one point for ICH volume >30 ml, one point for intraventricular extension of ICH, one point for a GCS of 5–12, and two points for a GCS of 3–4.

We then created a New ICH score by adding an additional point for the use of OAC. The maximum New ICH score was 7.

### Statistical Analyses

Categorical variables are presented as frequencies with percentage, continuous and ordinal variables as mean with SD or median with quartiles, depending on the distribution of the data.

First, we investigated whether OAC use is a predictor of 30-day mortality. We performed univariable logistic regression analysis with 30-day mortality as outcome variable and the variables in the ICH score (age, GCS, intraventricular extension, ICH volume, and infratentorial location) and OAC use as predictors. We then performed a multivariable logistic regression using the same variables to see whether OAC use is an independent predictor. There were no problems with multicollinearity.

Second, we investigated ICH score in both OAC-ICH and non-OAC-ICH. Correlation between ICH score and 30-day mortality was determined in patients with OAC-ICH and in patients with non-OAC-ICH, using Spearman correlation test. We computed receiver operating characteristic (ROC) curves and determined the area under the curve (AUC) in OAC-ICH and non-OAC-ICH.

Finally, we tested the New ICH score: Spearman correlation was determined between ICH score and mortality, and between New ICH score and mortality. We computed ROC curves and determined and compared the AUC for both ICH score and New ICH score. We also computed the difference in net benefit between ICH score and New ICH score at a threshold of 90% for predicted probability of mortality, which indicates how many more true positive mortality classifications can be made with the same number of false positive classifications.

Statistical analysis was conducted using IBM SPSSS statistics 22.0. Analysis of net benefit was performed in R version 3.1.1. A *p* value of <0.05 was considered significant.

## Results

We recorded 1,276 consecutive patients with a spontaneous non-traumatic ICH. Of those, 44 (3%) were excluded due to incomplete data on ICH score or 30-day mortality status, which left 1,232 patients in the analyses. Table 1 presents the characteristics of the included patients. Mean age was 73.0 (SD 12.5) years, and 641 (52.0%) were male. 30-Day mortality was 39.3% (*n* = 484). There were 282 (22.9%) OAC-ICH. Median INR in these patients was 3.5 (IQR 2.7–4.4).

**Table 1:** Patient characteristics.

	All intracerebral hemorrhage (ICH) patients	OAC-ICH	Non-OAC-ICH	*p*Value
		
	*n* = 1,232	*n* = 282	*n* = 950	
Sex, male (%)	641 (52.0)	155 (55.0)	486 (51.2)	0.261
Age, years (SD)	73.0 (12.5)	77.1 (8.3)	71.7 (13.3)	<0.001
Age, >80 (%)	394 (32.0)	117 (41.5)	277 (29.2)	<0.001
INR (IQR)^a^	1.0 (1.0–2.2)	3.5 (2.7–4.4)	1.0 (1.0–1.1)	<0.001
Intraventricular extension (%)	526 (42.7)	143 (50.7)	383 (40.3)	0.002
Infratentorial (%)	182 (14.8)	61 (21.6)	121 (12.7)	<0.001
ICH volume, cm^3^ (IQR)	16.0 (4.6–44.1)	20.1 (5.9–60.0)	14.3 (4.3–40.5)	0.001
Volume, >30 cm^3^ (%)	435 (35.2)	118 (41.8)	317 (33.4)	0.009
Glasgow Coma Scale (GCS) (IQR)	13 (10–15)	13 (8–15)	13 (10–15)	0.004
GCS 13–15 (%)	705 (57.2)	145 (51.4)	560 (58.9)	0.025
GCS 5–12 (%)	418 (33.9)	99 (35.1)	319 (33.6)	0.634
GCS 3–4 (%)	109 (8.8)	38 (13.5)	71 (7.5)	0.002
30-Day mortality (%)	484 (39.3)	155 (55.0)	329 (34.6)	<0.001

*^a^INR was available in *n* = 951 patients (OAC-ICH *n* = 270 and non-OAC-IC *n* = 681)*.

### OAC Use and 30-Day Mortality

Table 2 shows the associations between 30-day mortality and the different variables in the ICH score. All variables, including OAC use, showed a significant association with 30-day mortality. In multivariable analysis, OAC use was independently associated with 30-day mortality (OR 2.09, 95% CI, 1.48–2.95; *p* < 0.001).

**Table 2:** Univariable and multivariable logistic regression analysis for variables predicting 30-day mortality.

	30-Day mortality					
	Univariable analysis			Multivariable analysis		
	**OR**	**95% CI**	***p*Value**	**OR**	**95% CI**	***p*Value**
Age >80	1.89	1.48–2.41	<0.001	2.01	1.47–2.74	<0.001
Glasgow Coma Scale (GCS) 13–15	1.00			1.00		
GCS 5–12	6.59	5.01–8.65	<0.001	3.90	2.86–5.32	<0.001
GCS 3–4	63.85	29.01–140.54	<0.001	27.10	11.85–62.00	<0.001
Infratentorial	1.39	1.02–1.91	0.040	1.95	1.29–2.93	0.001
Intracerebral hemorrhage ICH volume >30	6.90	5.32–8.94	<0.001	4.03	2.93–5.55	<0.001
Intraventricular extension	5.13	4.01–6.57	<0.001	2.52	1.87–3.40	<0.001
OAC use	2.30	1.76–3.02	<0.001	2.09	1.48–2.95	<0.001

### ICH Score in OAC-ICH and Non-OAC-ICH

The median ICH score in non-OAC-ICH and OAC-ICH are 1 (IQR 1–3) and 2 (IQR 1–3), respectively (*p* < 0.001). 30-Day mortality in non-OAC-ICH was 329 (34.6%) and in OAC-ICH 155 (55.0%; *p* < 0.001).

There was a positive correlation between ICH score and mortality in non-OAC-ICH, Spearman’s rho 0.57, *p* ≤ 0.001. Correlation between ICH score and mortality in OAC-ICH was also significant, Spearman’s rho 0.56, *p* ≤ 0.001. The ICH score performed slightly better in non-OAC-ICH (AUC of the ROC curve 0.840; 95% CI, 0.812–0.867) than in OAC-ICH (AUC 0.816; 95% CI, 0.767–0.864), but this difference was not significant (*p* = 0.39).

### ICH Score and New ICH Score Including OAC Use

**Figure 1:**
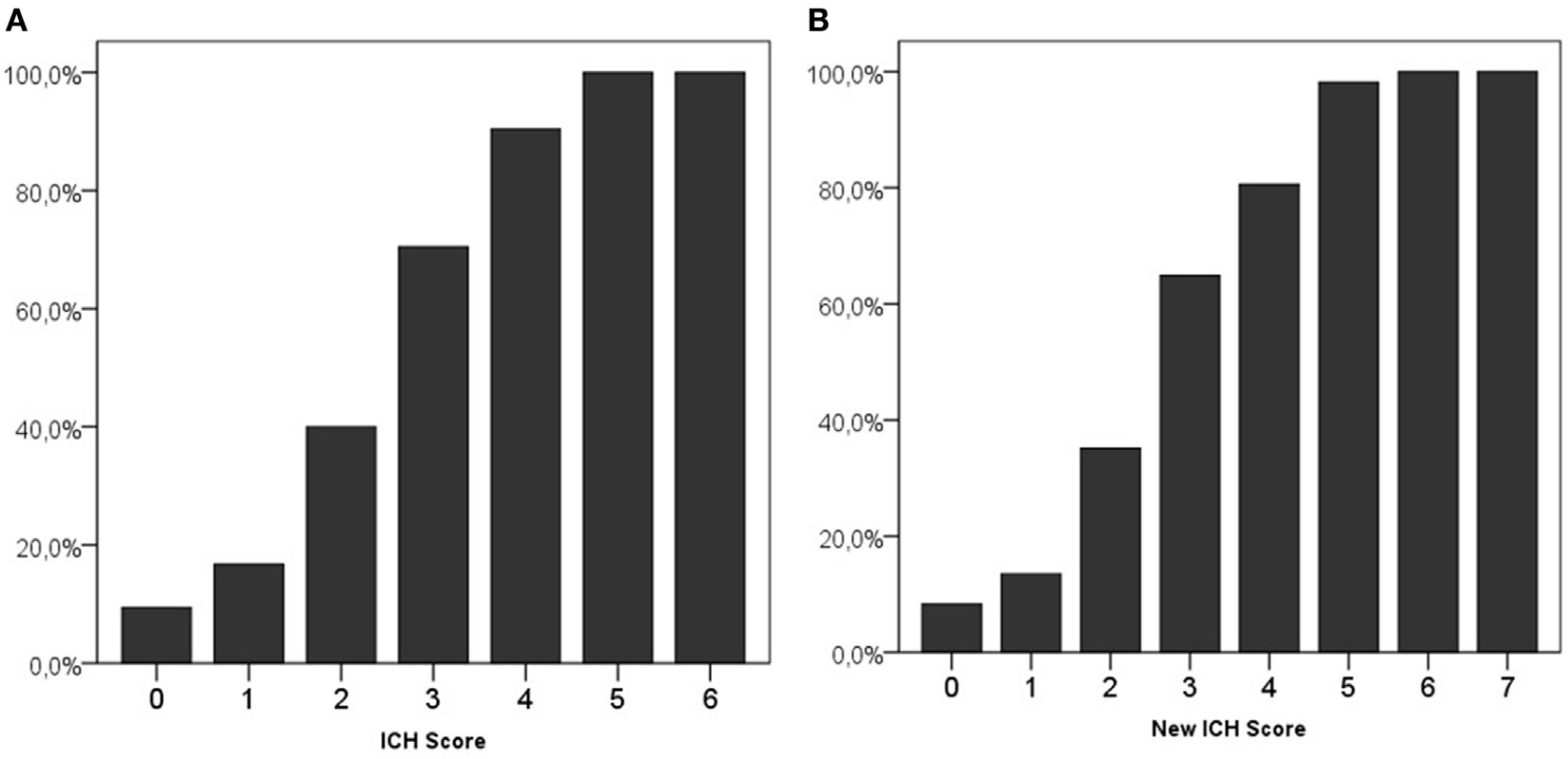
30-Day mortality on different scores of the intracerebral hemorrhage (ICH) score **(A)** and new ICH score **(B)**.

An ICH score of 0 was present in 265 (21.5%) patients, and 2 (0.2%) patients had the maximum ICH score of 6. Both patients with an ICH score of 6 used OAC and both died. Mortality in patients with different ICH scores is shown in Figure 1A.

Using the new ICH score, 229 (18.6%) patients had a score of 0, and 2 (0.2%) had the maximum score of 7. All patients with a New ICH score of 6 or 7 died. Figure 1B shows the mortality per category in the new ICH score.

The ICH score and the New ICH score were both significantly correlated with 30-day mortality (Spearman correlation coefficient rho 0.58, *p* < 0.001 and 0.59, *p* < 0.001, respectively).

**Figure 2:**
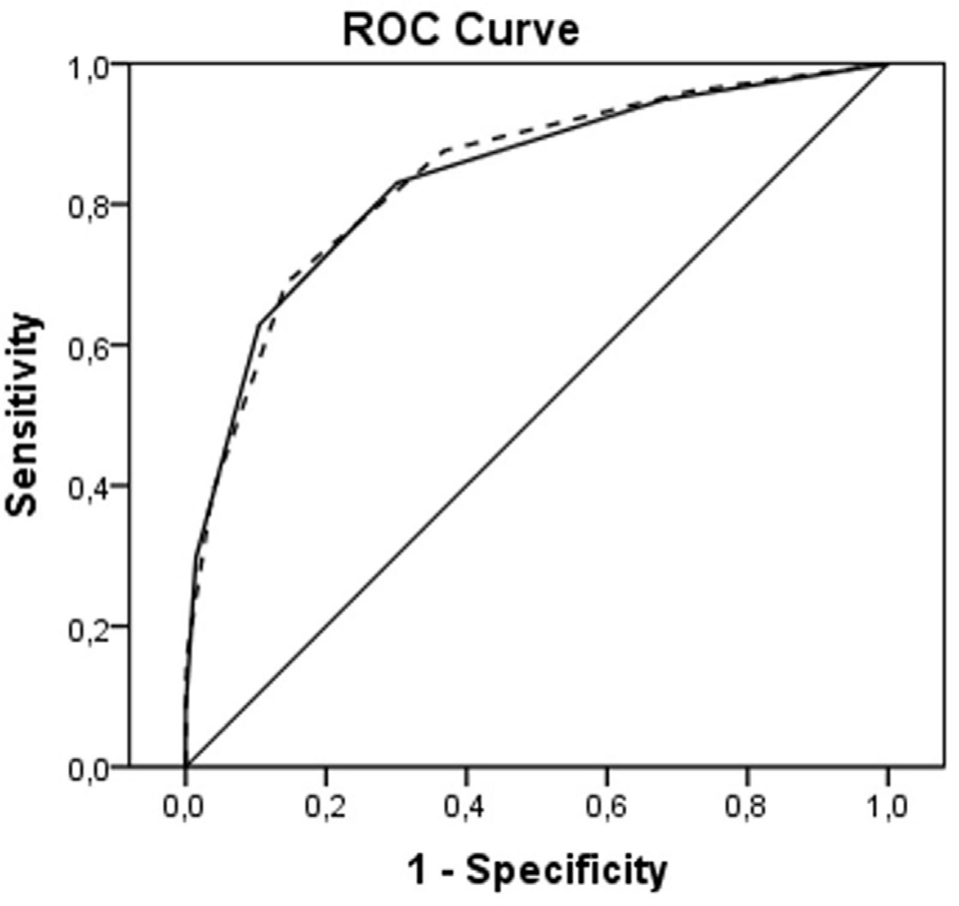
Receiver operating characteristic (ROC) curves for intracerebral hemorrhage (ICH) score (solid line) and New ICH score (dashed line).

**Figure 3:**
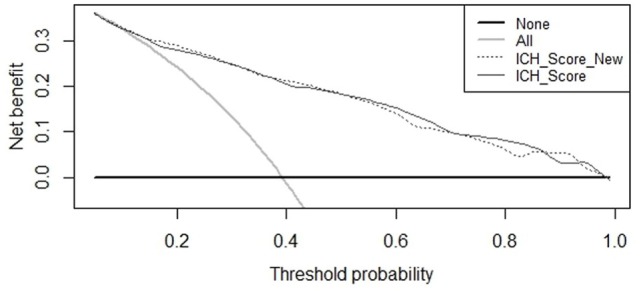
Net benefit curves for prediction of 30-day mortality by intracerebral hemorrhage (ICH) score and New ICH score.

Receiver operating characteristic curves are shown in Figure 2. The AUC for the ICH Score was 0.837 (95% CI, 0.813–0.860), the New ICH score had an AUC of 0.840 (95% CI, 0.817–0.863). The difference was not significant (*p* = 0.36). The New ICH score had a net benefit of 0.016 compared with the ICH score at a threshold probability of 90% (Figure 3). This means that the New ICH score can truly predict 90% mortality risk in 1.6% patients more than the ICH score, without any more untrue mortality predictions.

## Discussion

In this study, we aimed to investigate the performance of the ICH score in predicting 30-day mortality in OAC-ICH and non-OAC-ICH. ICH score performed equally well in both groups. Furthermore, OAC use was an independent predictor of mortality, but adding OAC use to ICH score did not improve prognostic performance.

Predicting an outcome prognosis in ICH patients is important as it guides clinical decision making. This accounts especially for OAC-ICH, because, despite reversal of OAC which is one of the few therapeutic options in ICH, mortality and disability are high. Our current understanding of the pathophysiology and the factors defining the outcome of OAC-ICH is still limited. Baseline ICH volume and GCS at presentation, factors which are incorporated in the ICH score, have been identified as independent predictors of mortality in both non-OAC and OAC-ICH (18). However, other factors that are more prevalent in OAC-ICH than in non-OAC-ICH such as comorbidities or hematoma and IVH expansion, may also be important determinants of prognosis (10, 13). Because these are not included in the ICH score, it might be that ICH score would perform less well in OAC-ICH compared with spontaneous non-OAC-ICH. We confirmed the performance of the ICH score to be comparable in OAC-ICH and non-OAC-ICH. Our results are consistent with results that were recently reported in a single-center, small (*n* = 170) study that evaluated hospital mortality (15). The results underline the usefulness of the ICH score. However, it should be noted that it was recently shown that early subjective clinical judgment of physicians correlated even more closely with 3-month outcome than ICH score (19) and that reassessment after 5 days of care can also improve the accuracy of prognosticating outcome in patients with ICH (20).

Multiple prognostic models (or scores) have been developed (2–8) to predict mortality and functional outcome after ICH. Although a substantial proportion of all ICH is related to OAC use, and OAC use is associated with increased mortality, it is striking that many prognostic models such as ICH score and the Essen score do not consider OAC use (2, 3). Cheung and Zou developed a modified ICH score and considered clotting time but it was not an independent predictor and it was therefore not included into the modified score (8). In the FUNC score, which was developed to predict likelihood of functional independence, use of warfarin was described in the baseline characteristics, but as it was not an independent predictor, it was not included in the final prognostic model (6). The EDICH grading scale included INR and showed predictive value for 30-day mortality in a small sample of 191 ICHs but has never been used nor validated in other studies (5).

In our study, we found OAC use to be a risk factor for 30-day mortality, independent of the five components that make up the ICH score (ICH volume, age, IVH extension, infratentorial location, and GCS). Despite this, adding OAC use to the ICH score did not improve the predictive value for mortality as shown in the ROC curves. However, comparison of ROC curves warrants some methodological reservations (21). It has been shown that an independent association of a new risk factor with an outcome often does not parallel into improved prediction in ROC curve beyond that of basic risk factors, especially when the AUC of the basic model already is high. This does not strike out the importance of the new independent risk factor. The net benefit of the New ICH score was, however, not very large compared to the ICH score in predicting a high risk of mortality.

Our study has limitations. First, we retrospectively analyzed data. Second, withdrawal of treatment could be an important confounder in the association with mortality; this affects almost all observational studies on outcome in ICH. However, we think it is unlikely that OAC use itself might have affected the decision of withdrawal of support. Third, we did not record functional outcome nor 1-year mortality. Although these are important outcome measures, ICH score was developed to predict 30-day mortality. Fourth, this study was performed before the era of direct OAC (DOAC) and only looked at vitamin K antagonists. The results can therefore not be generalized to patients with an ICH in the setting of DOAC use. Finally, it could be that anticoagulation level (INR) or reversal therapy is more important than OAC use *per se* (22). INR values were not available in all patients. In OAC-ICH patients in whom INR was measured, 91% had INR ≥2.0.

In conclusion, the ICH score remains a useful tool for predicting 30-day mortality in patient who use and patients who do not use OAC. The use of OAC is associated with higher 30-day mortality but adding OAC use to the existing ICH score did not improve the predictive value of this score.

## Ethics Statement

The medical ethics committee of Maastricht University Medical Center approved the study and waived the requirement for obtaining informed consent because it is a retrospective observational study.

## Author Contributions

JS, FS, RH, and RO contributed to the conception and design of the study. FS, KB, and DC collected the data. FS and RH organized the database. JS and RH performed the statistical analysis. JS and RH wrote the first draft of the manuscript. All authors contributed to manuscript revision, read, and approved the submitted version.

## Conflict of Interest Statement

The authors declare that the research was conducted in the absence of any commercial or financial relationships that could be construed as a potential conflict of interest.
